# Procurement of Pancreatic Tissue for Research From Deceased Donors Before vs After the CMS Final Rule in 2020

**DOI:** 10.1001/jamanetworkopen.2023.32395

**Published:** 2023-09-06

**Authors:** David S. Goldberg, Darren D. Lahrman, Matthew Wadsworth

**Affiliations:** 1 Division of Digestive Health and Liver Diseases, University of Miami Miller School of Medicine, Miami, Florida; 2 Life Connection of Ohio, Toledo

## Abstract

This cohort study examines changes in research pancreas procurement from deceased donors before and after the Centers for Medicare & Medicaid Services (CMS) updated its Final Rule in November 2020.

## Introduction

The Centers for Medicare & Medicaid Services (CMS) updated the Final Rule for organ procurement organizations (OPOs) in November 2020, creating a carveout whereby a pancreas or islet cells procured for research counts as a transplanted organ, and an individual who only donates a research pancreas and/or islet cells is a donor.^1,2^ We recently demonstrated a increase in the absolute number of pancreases procured for research, coinciding with the Senate Finance Committee’s investigation.^3,4^ Here, we sought to evaluate OPO-level changes in research pancreas procurement standardized to OPO volume and their associations with overall OPO performance (ie, CMS performance tiers).^5^

## Methods

This retrospective cohort study follows the Strengthening the Reporting of Observational Studies in Epidemiology (STROBE) reporting guidelines and uses Organ Procurement and Transplantation Network (OPTN)/United Network for Organ Sharing (UNOS) data from October 1, 2018, to March 31, 2023 (equal time before and after the rule change). We evaluated OPO data in 3-month blocks (ie, quarters [Q]) to avoid bias from small samples sizes using smaller time units (ie, months). We calculated OPO-level quarterly research pancreas procurement rates (QRPPRs), defined as research pancreases procured per 100 deceased donors,^3^ and assessed for statistically significant changes across 3-month blocks based on nonoverlapping 95% CIs in the QRPPR from 1 quarter to the next.^6^

## Results

**Figure 1.**
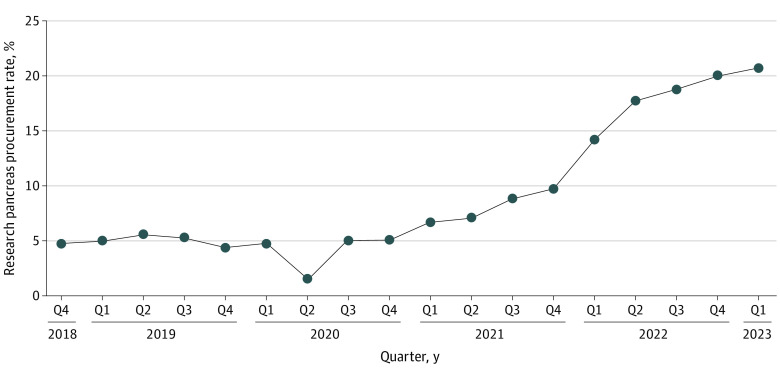
National Data for Quarterly Research Pancreas Procurement Rates, 2018-2023 Q indicates quarter.

Before the Final Rule, there were 1258 research pancreases procured (RPPs) among 27 144 donors (4.6 QRPPR), which increased to 4563 RPPs among 32 588 donors (14.0 QRPPR) after the rule. The national QRPPR was stable in the pre–Final Rule period (excluding a decrease in Q2 2020 corresponding to the initial period of COVID-19), began to increase Q1 2021, and was 4-fold higher by Q1 2023 (Figure 1).

**Figure 2.**
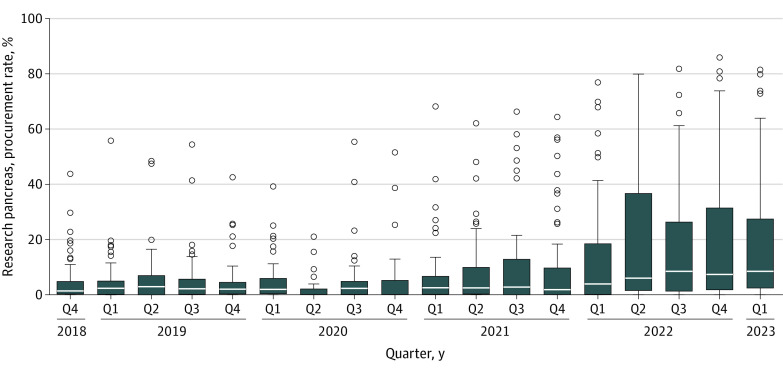
Box and Whisker Plot for Organ Procurement Organization–Level Quarterly Research Pancreas Procurement Rates, 2018-2023 Lines within boxes denote medians, tops and bottoms of boxes denote IQRs, error bars denote 95% CIs, and circles denote outliers. Q indicates quarter.

The change in OPO-level QRPPRs was not uniform across OPOs, with an increased range in OPO QRPPRs after passage of the Final Rule (Figure 2). For example, the number of OPOs with a QRPPR of 0 RPPs per 100 donors (ie, 0 research pancreases procured) decreased from 29 in Q4 2018 to 11 by Q1 2023, whereas the number with 20 or more RPPs per 100 donors increased from 4 to 16. Of the 16 highest research pancreas–procuring OPOs, 3 were tier 1 (highest-performing OPO; 15 total at tier 1) based on the 2023 CMS Interim Performance Report, whereas 7 of 18 were tier 2 and 6 of 23 were tier 3.^5^ There were 16 OPOs with a statistically significant increase in their QRPPR from 1 quarter to the next, of which 14 (87.5%) occurred after approval of the CMS Final Rule—6 were tier 3, 6 were tier 2, and 2 were tier 1.

## Discussion

This cohort study found that, since CMS updated its OPO Final Rule in 2020, there has been a rapid increase in procurement of pancreases for research. Although the rule’s intent may have been to encourage pancreas procurement for islet cell isolation, it is clear that tier 2 and 3 OPOs (those at risk for possible or definite recertification) were overrepresented among those with the biggest increases in these procurements.

The study is limited because OPTN/UNOS do not capture data on the research study or ultimate disposition of research organs (eg, islet cell isolation vs research unrelated to organ transplantation). Determining the validity of these research procurements is beyond the scope of this study.^3,4^ However, these pancreas research procurements have implications for CMS’s OPO recertifications in 2026 because (1) categorizing research pancreases as transplanted organs and research pancreas–only individuals as donors are not equivalent today to an organ that is transplanted, and (2) it impacts CMS’s age-adjusted metrics. Although we applaud research efforts by OPOs, CMS should consider revising their Final Rule to consider research procurements as distinct from an OPO’s performance evaluation.

## Notes

Supplement.
Data Sharing Statement
