# Supplementary material for: Procurement of Pancreatic Tissue for Research From Deceased Donors Before vs After the CMS Final Rule in 2020
Source: JAMA Netw Open. 2023 Sep 6;6(9):e2332395. doi: 10.1001/jamanetworkopen.2023.32395 (PMC10483317; doi:10.1001/jamanetworkopen.2023.32395)
Supplement: Supplement. — Data Sharing Statement [file jamanetwopen-e2332395-s001.pdf]

Data Sharing Statement

Goldberg. Procurement of Pancreatic Tissue for Research From Deceased Donors Before vs After the CMS Final Rule in 2020. *JAMA Netw Open*. Published September 06, 2023. doi:10.1001/jamanetworkopen.2023.32395

Data
Data available: No

Additional Information
Explanation for why data not available: The data are publicly available but the DUA does not allow sharing of the data. We can share the source code upon request.
